# Towards an integrated clinical framework for patient with shoulder pain

**DOI:** 10.1186/s40945-018-0050-3

**Published:** 2018-05-30

**Authors:** Diego Ristori, Simone Miele, Giacomo Rossettini, Erica Monaldi, Diego Arceri, Marco Testa

**Affiliations:** 1 Via Veneto, 6, Subbiano, Arezzo Italy; 2 Via Paolo VI, Cologne, Brescia Italy; 3 Via de Gaspari, 9, Montecchio Maggiore, Vicenza Italy; 4 Via Italo Svevo, 2 Codogno, Lodi, Italy; 5 Via Eugenio Scalfaro, 17, Catanzaro, Italy; 6 Via Magliotto, 2 17100, Savona, Italy; 7 Department of Neuroscience, Rehabilitation, Ophtalmology, Genetics, Maternal and Child Health, University of Genova, Campus of Savona, Savona, Italy

**Keywords:** Shoulder pain, Diagnosis, Rehabilitation treatment, Clinical framework

## Abstract

**Background:**

Shoulder pain (SP) represents a common musculoskeletal condition that requires physical therapy care. Along the years, the usual evaluation strategies based on clinical tests and diagnostic imaging has been challenged. Clinical tests appear unable to clearly identify the structures that generated pain and interpretation of diagnostic imaging is still controversial. The current patho-anatomical diagnostic categories have demonstrated poor reliability and seem inadequate for the SP treatment.

**Objectives:**

The present paper aims to (1) describe the different proposals of clinical approach to SP currently available in the literature; to (2) integrate these proposals in a single framework in order to help the management of SP.

**Conclusion:**

The proposed clinical framework, based on a bio-psychosocial vision of health, integrates symptoms characteristics, pain mechanisms and expectations, preferences and psychosocial factors of patients that may guide physiotherapist to make a diagnostic triage and to choose the right treatment for the individual patient.

## Background

Shoulder pain (SP) is a common musculoskeletal condition that can influence negatively the function of the entire upper limb [1]. The prevalence of SP ranged between 7 and 26% within the general population, increasing with age [2]. Most of the patients affected by SP describe the symptoms as “troublesome pain” [3]. When these symptoms become persistent and recurrent, the demand for medical consulting increases [4].

A large number of diagnostic categories have been developed: they are based on patho-anatomical classifications, such as tendinopathies, bursitis, labral tears, tendon tears, impingement, etc. [5]. However, there is considerable uncertainty regarding these diagnostic criteria [6], and the basis for them has been repeatedly challenged [7, 8]. Clinically, it may not be possible to distinguish between these patho-anatomical diagnostic categories with certainty [9].

Schellingerhout [6] defined the shoulder classification process as “a Babylonian confusion of tongues and seem to be of little benefit for those with SP”. This conclusion is in line with Buchbinder [9]: analyzing 5 classification systems based on patho-anatomical way of soft tissue disorders, she argued that they may not be acceptable for lack of validity and reliability of the inclusion criteria that create an overlapping of categories.

As consequence, we have thought it would be clinically useful to overcome the diagnostic difficulties by proposing a new pragmatic and symptoms-based model, coherent with a bio-psychosocial approach and closer to patient’s needs. Moving from this vision, this debate aims to: 1) describe the reasons for this diagnostic inconsistency; 2) present the different alternative proposals existing in the literature; 3) integrate the different proposals in a single framework, in order to provide physiotherapists with a helpful tool to deal with SP patients.

## Is the usual diagnostic process of SP valid and helpful?

In clinical practice, the assessment of a patient with SP is based upon an in-depth conversation (relevant history taking, understanding the patient’s complaints, and defining his/her psychosocial status) and a clinical assessment, which, in some cases, may be supported by imaging (e.g. magnetic resonance imaging [MRI] or ultrasound [US]). This approach is designed to enable a clinical diagnosis useful to guide the subsequent physiotherapy treatment [10].

### Importance of clinical physical tests in SP

Physical tests are tools commonly used in clinical practice, created to help the physiotherapist to identify which anatomical structures are involved with the patient’s symptoms. They are non-invasive, quick, convenient, and provide immediate results [11]. However, their interpretation may differ with the examiners’ clinical expertise [11].

#### Anatomical basis

Green et al. state that only few studies give information concerning the anatomical basis of the proposed tests [12]. Only four tests among those included in their review present a clear anatomical base. For these reasons, the author suggests a lack of assumptions in order to know what is happening in the shoulder during these assessment procedures [12].

The Hawkins-Kennedy represents a well-fitting example of the confusion surrounding the anatomical construct of these tests. It has been developed to identify the presence of sub-acromial impingement [13]. During years, many hypotheses have been suggested: compression of supraspinatus tendon under the coraco-acromial ligament [13], compression of the structures of the sub-acromial space between the head of the humerus and the acromion [14], contact between acromion and coraco-acromial ligament [15], compression of the long biceps head tendon [16].

#### Clinical usefulness

Others researchers have questioned the clinical usefulness of the physical tests in SP. Hegedus et al. have published three literature reviews discussing this topic [17–19]. They concluded that clinicians cannot confirm a diagnosis of the different shoulder problems neither with individual tests nor with cluster tests [18, 19]. They defined impingement as an “all-encompassing term” often meaningless with respect to the treatment [17]. Therefore, the clinical history collected from the patient and expert clinical reasoning seems to be crucial in the diagnostic process [19]. Hanchard et al. investigated physical test for impingement and associated lesions. Authors concluded that the body of evidence is extremely heterogeneous both in terms of performance (e.g. reliability, specificity and sensitivity) and relative interpretation thus making impossible to perform a synthesis of available data and to draw conclusions about their clinical application [11]. Furthermore, the reliability of these procedures was also found to be poor, with the authors concluding that there was a need of a new system of assessment in order to classify patients with SP [20].

### Importance of diagnostic imaging in SP

In clinical practice, diagnostic imaging (e.g. MRI, magnetic resonance arthrography [MRA], US and radiographs [X-RAY]) is considered to play an essential role during the assessment of patients with musculoskeletal disorders [21]. They are used both in specialist consultation (e.g. MRI and X-RAY to quantify the lesions and to support surgical planning) and in general practitioner (GP) consultation of primary care (e.g.US) [21]. Morphological and degenerative alterations are commonly considered relevant and together with patient’s history and examinations findings could support the choice of treatment [22].

#### Diagnostic accuracy

Lenza et al. [21], stated that MRI, MRA and US, are useful tools to identify massive rotator cuff tears in a population of patients included in a waiting list for surgery. Diagnostic performance of imaging decreases in line with the reduction of size the lesion. Moreover, the available studies generally present weak methodological quality and heterogeneity of the included populations. Indeed, the diagnostic accuracy of these tools dramatically decreases when applied in populations with poorly defined clinical features of association between structural lesions and symptoms [23].

#### Clinical usefulness and relevance

In 2013 a Cochrane editorial debated the diagnostic accuracy of imaging [24]; it was argued that the presence of asymptomatic rotator cuff tears [25] represents the “elephant in the room” responsible to challenge the relevance of diagnostic imaging. Some observational studies confirm this perspective: Girish stated that up to 2/3 of people with a rotator cuff lesion are asymptomatic [26] and rotator cuff tears are common in symptomatic and asymptomatic populations [27].

**Fig. 1:**
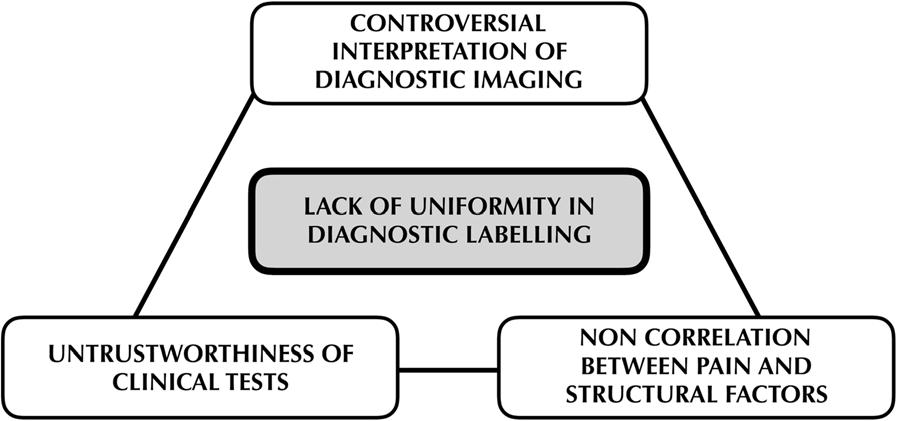
*Inconsistency of diagnostic labels in SP*. The weak correlation between structural factors and shoulder pain, together with the limited diagnostic value of bio-imaging and clinical tests, caused a lack of uniformity in diagnostic labelling

Usually, in patients with SP, there is uncertainty concerning the cause of pain and which risk factors are relevant to the onset of symptoms. Some authors suggest that the possibility of symptoms increases with the size of rotator cuff tear [28], while other authors proposed that the development of symptoms is mostly correlated to other non-structural factors, such as gender, age and psychosocial factors [29, 30]. Thus, clinical interpretation of diagnostic imaging in patients with SP remains controversial, suggesting that the biomechanical classification system is unsuitable (Fig. 1)*.*

## Are integrative procedures of assessment available?

The lack of reliability of clinical tests and limited usefulness of diagnostic imaging led some authors to suggest the integration of different assessment strategies, more pragmatic and focused on the results of functional assessment [31–33]. The existing proposals include: the shoulder symptom modification procedure (SSMP) [31], the staged approach for rehabilitation classification: shoulder disorders (STAR-shoulder) [32] and the Klintberg proposal [33] that synthesize a consensus statement of several shoulder rehabilitation experts (see Table 1).

**Table 1:** *Characteristics of existingproposals of assessment strategies*

Existing proposals of assessment strategies		
SSMP	Star-shoulder	Klintberg’s clinical algorithm
The SSMP is a series of clinical procedures aimed to reduce the patient’s symptoms. A procedure able to eliminate/reduce the symptoms is adopted as a treatment technique. If following the application of the SSMP, symptoms have not completely disappeared an exercise program is required; the SSMP is typically embedded within a graduated shoulder exercise program. Lewis suggests to apply the different techniques of the SSMP after the conduction of a preliminary assessment (composed by detailed history, screening for potential red-flag, functional/disability questionnaires administration, evaluation of impairments and if necessary orthopaedic tests and imaging).	The authors created a model providing a sub-classification of patients on the basis of patho-anatomical features, tissue irritability and individual impairments. Three steps are proposed: 1) screening, 2) patho-anatomical diagnosis (e.g. sub-acromial syndrome, frozen shoulder, glenohumeral instability) and 3) a rehabilitative step, based on the level of irritability.	The algorithm encompasses the functional assessment of a range of motion (ROM) and the evaluation of presence/absence of abnormal scapulohumeral motion pattern in order to identify patients with limited passive ROM or with reduced muscle performance that can be treated with specific exercises or manual therapy. The algorithm helps clinicians to choose the adequate therapeutic approach. Moreover, it allows flexibility during the assessment process. Algorithm-based re-assessment of the patients allows monitoring whether the proposed exercises are correctly targeted towards the prevalent impairment or is necessary to test other clinical adjunctive problems.

## Is it the time to move towards an integrated clinical framework for the assessment and treatment of SP?

These current approaches have evolved as a consequence of the uncertainty in biomedical model, but they still present some limitations that reduce their ability to interpret comprehensively all the features of SP. The STAR-shoulder classification and SSMP do not assess the contribution of central sensitization (CS) [31, 32]. Klintberg et al., propose a pure mechanist approach based on a diagnostic algorithm for the assessment of movement patterns [33]. Moreover, excluding SSMP [34], the reliability of the existing proposals is still lacking.

**Fig. 2:**
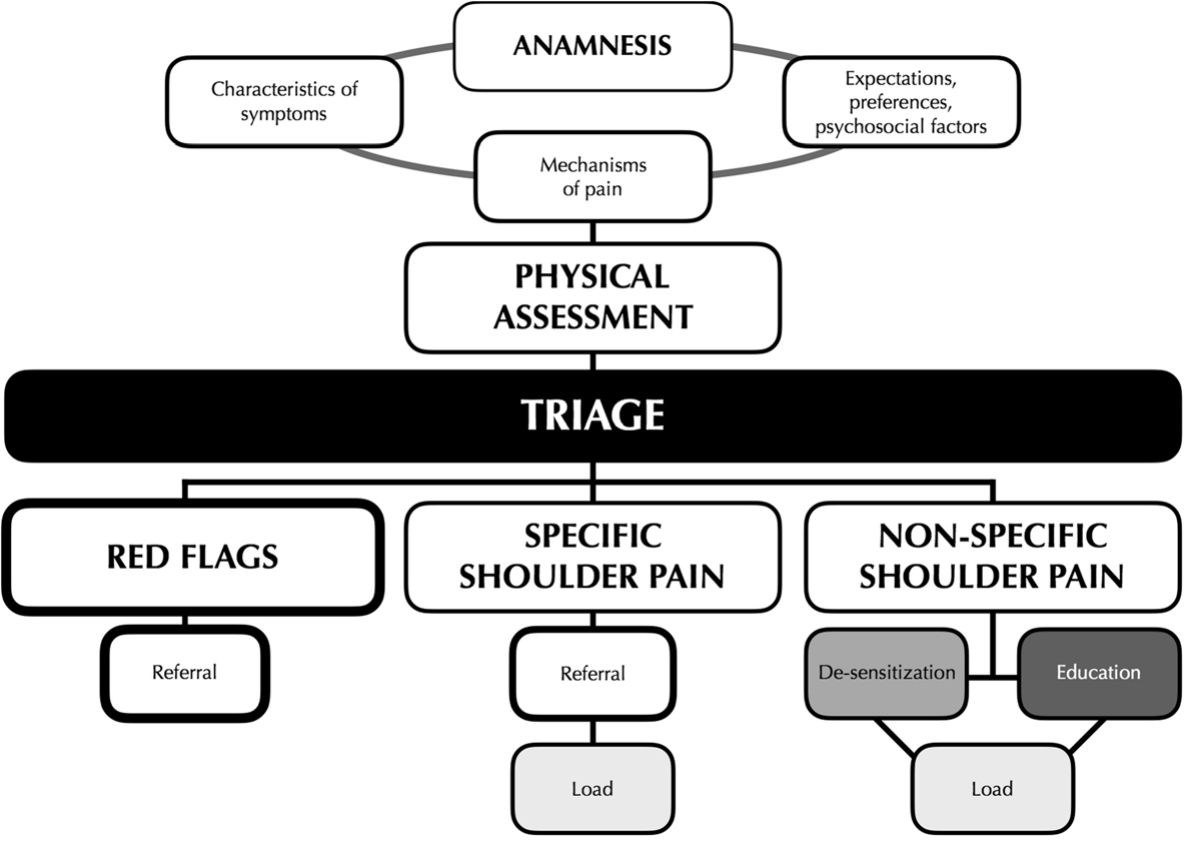
*The integrated clinical model for the assessment and treatment of SP.* By history taking, the physiotherapist investigates pain characteristics, its prevalent mechanisms and patient’s beliefs and expectations. Integrating this information with the results of the physical assessment, the physiotherapist classifies the shoulder pain condition with three diagnostic labels: Red Flags and Specific SP which require a referral to a specialist consultation and Non-specific SP which falls within the competence of the physiotherapist

Moving from the existing proposals, we have tried to overcome the limits of exclusively mechanistic classification and to create a framework for assessment and treatment of SP that integrates and includes them in a bio-psychosocial perspective (Fig. 2). Anamnesis, physical assessment, triage and treatment are the four clinical procedures mainly affected by the implementation of our clinical perspective.

### Anamnesis

The anamnesis is a milestone in the assessment of patients with musculoskeletal dysfunction [35]. Different anamnestic elements must be collected (e.g. characteristics of symptoms, mechanisms of pain, expectations, preferences and psychosocial factors of patients), weighted and included in the clinical reasoning process to guide the subsequent physical examination [11, 21].

#### Characteristics of symptoms

Specific information regarding impairments and symptoms of SP must be investigated during the colloquium with the patient, including; onset, quality, 24 h behavior, localization, alleviating and aggravating factors [35]. Moreover, there is a need to consider symptoms in other body segments reported by patients correlated with the main problem (e.g. cervical or thoracic spine), the comorbidities, the previous consultancy with other healthcare professions (e.g. orthopaedic), the previous positive/negative experiences with a specific therapeutic approach, the patient’s life context (e.g. family or work problems) and the screening of red flags [35]. Physiotherapists should also investigate the limitation of activities and restriction of participation associated with SP impairments with specific questionnaires (e.g. the disabilities of the arm, shoulder and hand questionnaire [DASH], shoulder pain and disability index [SPADI], the pain self-efficacy questionnaire [PSEQ]) [36–38].

#### Mechanisms of pain

The features of symptom help physiotherapists to understand the underpinning mechanism behind patient’s pain presentation, such as nociceptive pain (NP) or CS. Potential indicators of NP are: the localization of pain in the area of injury/dysfunction, the description as intermittent and sharp during movement or constant dull or throbbing ache at rest, as well as a clear response consistent with aggravating and easing mechanical factors [39].

Evidence suggests that CS phenomena are present in patients with SP [40, 41]. Potential indicators of CS are: the absence of correlation or inconsistency between provocative stimulus and response, the discontinuity of pain, its unpredictability and increase with non-specific movements, the variable anatomical distribution, the non-coherence of any referred pain and the widespread irritability. Moreover, other elements are disturbed sleep, areas of numbness, misperception of the affected segment, the feeling of swelling in absence of evident edema which may increase with closed eyes [39, 42].

In addition to the anamnestic elements, physical signs of CS must be investigated during physical examination (e.g. swelling, weakness or stiffness of the affected segment, lack of correspondence between specific movements and pain) [42]. During the assessment process, the identification of pain mechanism on the basis of patient’s dysfunction could help physiotherapists to better manage the SP and to target more adequately the treatment [43].

Unfortunately, there is only preliminary discriminative validity of mechanism-based classification of musculoskeletal pain [39]. The use of some self-reported tool (e.g. central sensitization inventory [CSI]) could be useful for physiotherapists to quantify symptoms severity of CS, thus guiding the clinical reasoning process [44].

#### Expectations, preferences and psychosocial factors of patients

During the anamnesis, it is essential to investigate the patient’s expectation, preferences and the presence of psychosocial factors (yellow flags) in order to guide the subsequent best treatment decisions and to reduce the patient’s risk for developing long-term disability [45].

Recent evidence suggests that expectations and preferences about the physiotherapy treatment play an important role as influencers of musculoskeletal outcomes [46, 47] also in patients with SP [48]. It is useful to ask the patients what he expects from physiotherapy to understand if the achievement of the desired outcomes is possible or not. The physiotherapists should also investigate the patient’s preferences towards a specific treatment (e.g. manual therapy or exercises) [46, 47].

Moreover, the predominance of specific psychological factors such as personal and environmental elements must be analysed. The physiotherapist should screen for older age (more of 50 years), higher perceived pain intensity, longer duration of symptoms, previous injury, extensive sick leave, unemployment, co-morbidities, previous SP, poor perceived general health, avoidance of activity for fear of pain and harm, perceived high job demands and low job satisfaction, higher body mass index, poor social support, personal problems (alcohol, financial, marital) [49, 50].

### Physical assessment

The evaluation of the quality of active and passive shoulder and cervical spine movements [51], the range of motion (ROM) and the shoulder muscles strength are the priority of the assessment [33]. Physical assessment should explore the provocative movements of the patient’s pain. It can be an active or passive movement of the shoulder [31] or a movement of the neck region (in this case a comprehensive clinical assessment of this region should be considered) [20].

Physical assessment is also aimed to confirm the presence of CS signs suspected during anamnesis, such as swelling, weakness or stiffness of the affected segment, lack of correspondence between specific movements and pain [39, 42], while the clinical utility of quantitative sensory testing (QST) for the detection of CS is questionable and still a source of debate [52].

### Triage and treatment

From a clinical-pragmatic perspective, there is the need to modify the diagnostic labeling used for patients with SP [6, 17, 20]. As proposed for other body regions (e.g. lumbar spine, cervical spine), also in the shoulder complex, there is a growing awareness of the very limited ability to identify a specific structure responsible for the patient’s symptoms [53]. Therefore, a transition of the SP assessment from a strictly mechanistic to a more bio-psychosocial oriented approach seems necessary. The analysis of the patient’s history, beliefs, preferences and functional movements, have recently assumed a key role [54].

In our clinical framework, we propose a process of diagnostic triage that adopts a classification system similar to the one already adopted for other regions (e.g. lumbar spine [55, 56]): *red flags*, *specific pain*, *non-specific pain*. Firstly, the physiotherapist should exclude Red Flags, then distinguish patients, classifiable as *specific shoulder pain*, with signs and symptoms of musculoskeletal dysfunction for which is necessary the referral to an orthopaedic evaluation before establishing a physiotherapy treatment [57, 58]. Finally, the physiotherapist can classify as *non-specific shoulder pain* [56] the patients presenting clinical features that do not belong to the two categories described above.

#### Red flags and specific shoulder pain

*Red flags* are sign and symptoms alerting the physiotherapist on a possible presence of a non-musculoskeletal, life-threatening pathology, fracture, infection, tumor and inflammatory rheumatic conditions [59]. However, physiotherapists must be careful in the evaluation of signs and symptoms of patients [60]. The prevalence and incidence of red flags in shoulder disorders are unknown [59, 61], thus limiting the identification of serious non-musculoskeletal pathology at the first consultation [62]. *Specific shoulder pain* indicates that symptoms could refer to a pathology that has a clear structural, patho-anatomic or pathophysiologic origin (e.g. symptomatic rotator cuff tears, superior labral tear from anterior to posterior [SLAP] or instability). It requires referral to an orthopaedic specialist to clarify diagnostic aspects or surgical needs [63, 64]. Signs and symptoms characteristic of these two categories are listed in Table 2. It is not necessary that all symptoms have to be present at the same time to guide physiotherapists during their clinical reasoning process [65–68]. When a conservative approach has been chosen for *specific shoulder pain*, physiotherapists may refer to specific options for treatment available in literature (e.g. for conservative treatment of patients with massive rotator cuff tears, we could propose stretching, proprioceptive and active exercises towards functional movements, increasing progressively the position of execution and the resistance) (see Table 4) [57, 58].

**Table 2:** *Red Flags and symptoms of specific shoulder pain*

Anamnestic and clinical Features of red flags	Sign and symptoms of Specific shoulder pain
Fever, shivering, changes in body temperature overnight, diaphoresis, nausea, unexplainable sweating overnight, vomiting, sphincteric complaints, diarrhoea, paleness, fatigue, lurching, fainting, exhaustion, excessive and unexplainable weakness, not linked to any physical effort, unexplainable loss of weight, skin rash, unexplainable multiple hematoma, lumps over the body, deformities, inability to lay supine in bed, marked muscle weakness, marked restriction of movement, limb atrophy, local pain and pain during load when age is less than 20 years old and more than 50.	Recent trauma of the shoulder complex, high reactivity of symptoms, pain during the night, limitation of flexion (< 90° both passive and active), apprehension, fear of movement and/or weakness during humeral external rotation.

#### Non-specific shoulder pain

Once the patient is categorized as *non-specific shoulder pain* [69]*,* the physiotherapist should recognize what is the prevalent mechanism of pain elaboration of the patient, and identify what are the functional movements that provoke symptoms. If the patient does not recognize precisely the pain provocative shoulder movements, physiotherapists can use shoulder orthopaedic tests to provoke pain [31]. Three strategies, overlaid and fused in every intervention of our clinical practice, should be adopted to treat *non-specific shoulder pain* patients: education, de-sensitization and load management. Overall, in the choice of treatment, the physiotherapist must integrate, as much as possible, expectations and preferences of patients thus adopting any previous positive physiotherapy solutions and avoiding the past negative experiences [46, 47].

##### Education

It is important to inform patients about their clinical condition, avoiding an excessive biomedical terminology (e.g. “shoulder impingement”), explaining the pain mechanisms underpinning their symptoms, their favorable prognosis, the strategies of treatment that are intended to use proprioand the value of self-management and home exercise [48, 70, 71]. This education process should be promoted throughout the whole treatment, thus enhancing the patient’s engagement and empowerment [70, 72]. In presence of high predominance of yellow flags, the patient should be monitored and educated, thus modifying any dysfunctional beliefs and overestimated expectations about SP and reconceptualising on a cognitive level any fear, harm and avoidance about shoulder activity [73].

##### De-sensitization

Manual therapy is one of the possible interventions to reduce SP. Mechanical stimuli applied to the skin of the patients by manual therapy, determine several neurophysiological mechanisms (e.g. peripheral, spinal and supra-spinal) that improve pain. Because of this variety of effects sources, we can assume that manual therapy can be considered as a therapeutic intervention able to de-sensitize the neurologic system that supports pain perception [74–77]. Manual therapy could play an important role to decrease fear of movement and catastrophization [75]. The physiotherapists should consider also the adoption of drugs (e.g. pain killers) or exercises to reduce pain and to desensitize the patient [51, 57], thus reducing the possibility of CS [78].

##### Load management

We considered as load every movement that could increase the ability to perform a limited/painful movement. The load is usually administered through exercise, thus playing an important role for this category of patients with SP [79]. Indeed exercises have both the capacity of re-conditioning the anatomical structures, with an effect on NP mechanisms [57, 80, 81] and the capacity of modulating the patient’s pain with an action on CS mechanisms [82]. This effect of movement and exercises have been demonstrated also in other body regions [81, 83]. Various load strategies will be described in the section below.

##### Algorithm of treatment

**Fig. 3:**
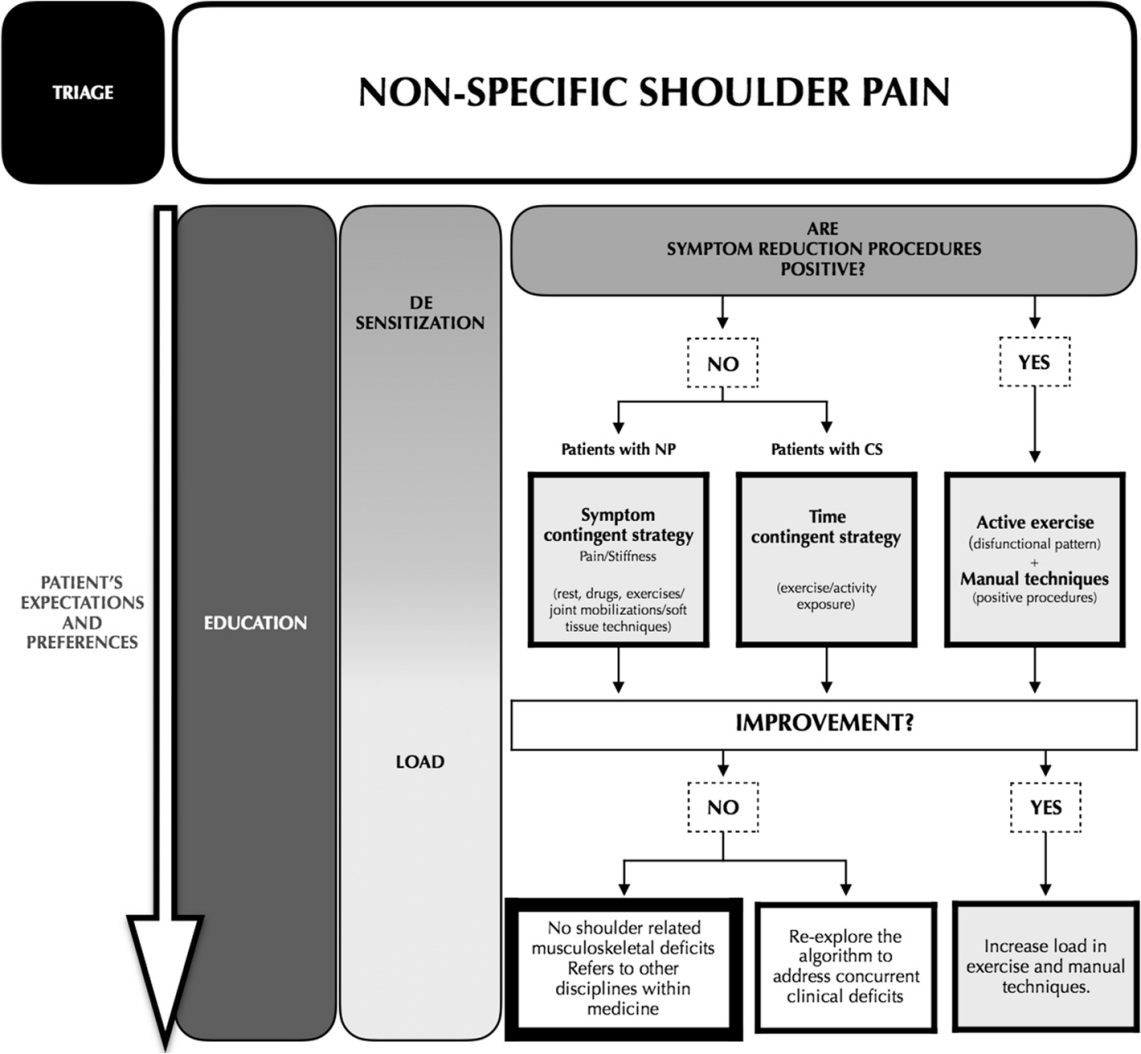
*Non-specific SP: the algorithm of treatment*. De-sensitization procedures should be adopted first. If an improvement of pain and/or patient’s satisfaction is obtained, the treatment load should be increased by using the positive procedures and specific exercises. If this first approach does not reach its goal, then therapeutic strategies based on the prevalent pain mechanism should be implemented. Symptom-contingent strategy or manual techniques (in cases of joint stiffness) and time-contingent strategy have to be used in patients respectively with prevalent NP or CS mechanisms. In case of lack of improvement, the patient should be re-assessed or referred to the specialist

To organize the treatment of patients with non-specific shoulder pain provoked by shoulder movements, is advisable to adopt the same functional approach proposed by Klintberg et al., that seems to be flexible and easy to perform [33] (Fig. 3). Once the painful movement is identified, the patient rates his/her pain on a numeric rating scale and then the physiotherapist attempts to modify it applying specific procedures [31, 57, 84–89] (see Table 3).

**Table 3:** Examples of diagnostic/therapeutic procedures to reduce the patient’s symptoms

Example of procedures for symptoms reduction	
SSMP (thoracic kyphosis, humeral head procedures, scapular position)	
Mulligan’s techniques of mobilization with movement	
Scapular assisted test and scapular repositioning test	
Manual or dry needling treatment of myofascial trigger points (mTrPs)	
Manual treatment of cervical and thoracic joints (mobilization/manipulation)	

Different procedures are administered to the patient until he/she reports a satisfying improvement occurring during or after the intervention [31]. Some authors stated that inter and intra-treatment changes may be predictive of improvement of the specific symptom as well as of the general condition of the patient: this phenomenon can be of support for the clinical decision-making [90, 91]. If the procedures of symptoms modification result effective, manual treatment in association with exercises (with a progressive amount of load based upon the clinical evolution of the patient) are adopted [51, 57, 85]. The pain-free therapeutic window identified by the positive response of symptoms modification procedures can be used to propose pain-free exercises (adopting positive procedures as exercises), regardless of the presence of CS component of the clinical scenario (see Table 4).

**Table 4:** *Load strategies for specific and non-specific shoulder pain*

Load strategies			
Specific shoulder pain	Non-specific shoulder pain		
	Responsive (NP or CS)	Non responsive NP	Non responsive CS
*Aim:* Increase movement/strength and flexibility. *What to do*: symptoms-contingent strategies; stretch, proprioceptive and active exercises progressively changing the position of execution and increasing number of repetition and the resistance according with patient’s pain.	*Aim:* reduce pain, fear, increase movement/strength. *What to do*: symptoms-contingent strategies; commute positive procedures in exercises plus load the dysfunctional pattern of patient’s movement increasing and modulating number of repetition and resistance according with patient’s pain.	*Aim:* reduce pain, increase movement/strength. *What to do*: symptoms-contingent strategies; stretch and active exercises progressively changing the position of execution and increasing number of repetition and the resistance according with patient’s pain.	*Aim:* reduce pain, fear, increase movement/strength. *What to do*: time-contingent strategies; graded exposure/activity starting from the identification of painful tolerated exercise in terms of number of repetitions and pain granted during the execution.

Regarding load management, the physiotherapist should play with an accurate tuning of the posology, in terms of specific target and modalities of execution (see Table 4) [32, 33, 43, 57, 79, 92, 93].

Even if some authors choose the structure(s) to which target the exercises on the base of the effect of the previously performed procedure (e.g. gleno-humeral muscles if humeral head and muscular conditioning procedures were positive; or thoracic spine muscles if modification of thoracic kyphosis resulted positive) [33, 57, 87, 93], according with Littlewood et al. [79] we suggest as preferable to rely on the dysfunctional pattern of movement of the patient in order to restore the activities of daily life (e.g. respecting the tolerance of movement in terms of pain/fatigue, if abduction is the painful/limited movement, the physiotherapist will increase and modulate number of repetition and resistance in abduction exercises).

If symptoms reduction procedures resulted negative without influence patient’s SP, the load will be managed by exercise, according with prevalent mechanisms of pain elaboration showed by the patient:

- Patients with NP mechanisms: if the clinical condition is characterized by high level of reactivity, the therapeutic approach will be based on patient education [32], de-sensitization with rest, drugs (refer to medical management) or graded motor imagery (GMI) [94]. In the more active treatment phase, once the reactivity is reduced, load management is predominant and exercises are proposed with the adoption of a “symptoms-contingent” strategy (the presence of symptoms limits the performance of exercises), targeting the dysfunctional motor task (e.g. a program of exercises that aim to load the impaired movement, starting from pain-free, simple, with low resistance exercises toward more complex functional-tasks) (see Table 4) [32, 33, 57, 80, 93, 95];
- Patients with CS mechanisms: de-sensitization and load management are coupled with the therapeutic approach. The clinical conditions with prevalent CS features are managed by patient’s education and “time-contingent” exercise (exercises have to be performed for a certain time, agreed with the patient, despite the presence of symptoms) in order to restructure the patient belief of association between pain-danger-harm (e.g. graded exposure/activity starting from the identification of painful tolerated exercise in terms of number of repetitions and pain granted during the execution) [42, 43, 70, 73, 82, 96–98]. Communication with patients (including information, reassurance and education) could also help exercises and it plays an important role in the achievement of this aim [99]. GMI and low intensity, aerobic/non-specific exercise also seems to be particularly useful in this category of patients [100] (see Table 4).

Concerning the prognosis, when a positive progressive improvement is obtained, an exercise training of 12 weeks duration is recommended [33, 92]. Moreover, the presence of a lower baseline pain and disability, a patient expectation of a ‘complete recovery’ as ‘a result of physiotherapy treatment’ in comparison to ‘slight improvement’, a higher pain self-efficacy and lower pain severity at rest enhances the likelihood to reduce pain and improve disability in SP [101]. Opposite, when capsular stiffness is a predominant feature of the clinical scenario, a longer time is needed to fully restore functional movement [95]. The concomitance of higher level of depression symptoms, catastrophizing thoughts, fear of movement, fear of pain and anxiety were related to higher disability, greater pain severity, lowest perceptions of clinical improvement and increased possibility of developing a pattern of CS in patients with SP [102–105]. Finally, in the short/medium term, the expected results are not reached, the patient should be re-evaluated re-exploring the different steps of the framework or referred to other specialists (e.g. orthopaedic) [33].

## Study limitations

In this paper, we adopted an evidence-based approach to guide physiotherapists in the management of SP, thus proposing a framework inspired by the bio-psychosocial model [106] and aligned to the International Classification of Functioning, Disability and Health [107]. Despite our best efforts, several limitations affected the suggested framework: 1) it is based on a discretionary expert opinion; 2) it is created without an international expert consensus methodology (e.g. Delphi study); 3) the selection of relevant articles was based on narrative review instead of a declared approach (e.g. systematic review); 4) its applicability, efficacy, validity and reliability has not been tested. Moreover, we actually could not classify different profile of patients under the label of non-specific shoulder pain [108]. When it will be possible, as previously happened for low back pain, it could permit us to optimize diagnostic and therapeutic proposals.

## Conclusions

Existing literature underlines the limits of a strictly anatomical model for the evaluation of patients with SP. The integration of the alternative purposes in that clinical framework could help to orientate physiotherapists towards a more bio-psychosocial and pragmatic approach. In the future, the category of non-specific shoulder pain and its peculiarities should be taken into account in diagnostic and prognostic research studies.

## Abbreviations

CS: Central sensitization
CSI: Central sensitization inventory
DASH: The disabilities of the arm, shoulder and hand questionnaire
GMI: Graded motor imagery
GP: General practitioner
MRA: Magnetic resonance arthrography
MRI: Magnetic resonance imaging
NP: Nociceptive pain
PSEQ: Pain self-efficacy questionnaire
QST: Quantitative sensory testing
ROM: Range of motion
SLAP: Superior labral tear from anterior to posterior
SP: Shoulder pain
SPADI: Shoulder pain and disability index
SSMP: Shoulder symptom modification procedure
STAR-shoulder: Staged approach for rehabilitation classification: shoulder disorders
US: Ultrasound
X-RAY: Radiographs
